# Ultralight, High Capacitance, Mechanically Strong Graphene-Cellulose Aerogels

**DOI:** 10.3390/molecules26164891

**Published:** 2021-08-12

**Authors:** Xiuya Wang, Ke Wan, Pengbo Xie, Yuanyuan Miao, Zhenbo Liu

**Affiliations:** Key Laboratory of Bio-Based Material Science and Technology of Ministry of Education, Northeast Forestry University, Harbin 150040, China; wangxiuya2019@nefu.edu.cn (X.W.); wkxlqy@nefu.edu.cn (K.W.); xiepengbo@nefu.edu.cn (P.X.); miao.yuanyuan@nefu.edu.cn (Y.M.)

**Keywords:** cellulose, graphene, aerogel, supercapacitor

## Abstract

With increasing energy demand driving the need for eco-friendly and efficient energy storage technology, supercapacitors are becoming increasingly prevalent in wearable devices because of their portability and stability. The performance of these supercapacitors is highly dependent on the choice of electrode material. The high capacitance and mechanical properties needed for these materials can be achieved by combining graphene’s stable electrical properties with renewable cellulose’s excellent mechanical properties into porous aerogels. In this study, graphene-cellulose hydrogels were prepared by a one-step hydrothermal method, with porous, ultra-light, and mechanically strong graphene-cellulose aerogels then prepared by freeze-drying. These composite aerogels possess excellent mechanical strength and high specific capacitance, capable of bearing about 1095 times the pressure of their own weight. Electrochemical tests show the specific capacitance of these composite aerogels can reach 202 F/g at a scanning rate of 5 mA/cm^2^. In view of their high surface area and fast charge transport provided by their 3D porous structure, graphene-cellulose aerogels have great potential as sustainable supercapacitor electrodes.

## 1. Introduction

Supercapacitors are an increasingly prevalent type of energy storage device, characterized by fast charge/discharge rates, long cycle life, high power density, and wide operating temperature range. They have established applications in numerous fields, including national defense, railways, new energy vehicles, electronics, communications, and aerospace [1,2,3]. Whether materials can be matched with practical applications in supercapacitors requires evaluating not only their electrical and mechanical properties, but within the broader context of high global resource consumption, their renewability and biodegradability must also be considered [4,5].

Graphene is an ideal electrode material for capacitor because of its large specific surface area, more energy storage per unit mass, fast charge-discharge, and theoretical capacitance of 550 F/g [6,7,8,9]. However, when graphene is used as electrode material alone, it is easy to stack and agglomerate due to van der Waals force and π–π interaction, which greatly reduces the specific surface area of the material. The specific surface area, pore structure, and electrical conductivity of the electrode material represented by graphene are the three key factors that determine the specific capacitance, power density, and energy density of the capacitor [10,11,12]. Thus, agglomeration is the primary technical barrier for the application of graphene materials in supercapacitors.

There are two main ways to solve graphene agglomeration and expand the application of graphene materials in supercapacitors. One is to prepare three-dimensional structures such as graphene hydrogel, graphene aerogel, and graphene foam [13,14]. The three-dimensional porous network of graphene can reduce the agglomeration of graphene, ensure the full contact between electrode and electrolyte, improve the mass transfer of electrolyte, and make it have excellent electrochemical performance [15,16,17]. Hydrothermal and chemical reduction methods are usually used to prepare cellulose hydrogel or aerogel. Compared with the chemical reduction method, the hydrothermal method has the advantages of being fast and simple while not requiring treatment of harmful residual reduction reagents and non-carbon impurities. Xu’s team proposed a one-step hydrothermal method to prepare graphene hydrogel, which is also the first report based on hydrothermal GO dispersion method to prepare restore oxidation graphene hydrogel [18,19,20]. The graphene hydrogel showed a specific capacitance of 175 F/g, which could be used as a good electrode material for capacitors. In order to further improve the electrical properties of graphene hydrogel, Shi et al. combined hydrothermal method and chemical reduction method [21]. The specific capacitance of graphene hydrogel can reach 220 F/g at a current density of 1 A/g. Since then, designing three-dimensional structures to improve the pore structure and electrical properties of graphene materials has become a new hot direction. The second method to solve the agglomeration of graphene and enhance the electrical properties is to incorporate other nanoparticles between graphene sheets to form graphene composite electrode. While reducing the agglomeration between graphene sheets, it can also maintain the large specific surface area of graphene. In the selection of spacers, cellulose has become one of the options with its advantages, including porous structure and hydrophilicity, which can promote the attachment of other materials to the fiber network structure [22,23,24,25,26,27,28,29]. At the same time, cellulose surface has many hydroxyl and hydrogen bonding sites, which can interact with other polymers to form a solid composite [30,31]. In addition, cellulose has the advantages of low density, renewable and biodegradable [32,33,34]. When it needs to be combined with other conductive materials, cellulose is used as a supporting substrate to make the composite exhibit excellent and stable properties [35]. Graphene has high conductivity, and there is no need for additives or adhesives when the composite material bound with cellulose is used as the electrode material of capacitors [36]. This simplifies the preparation process of the electrode and avoids the electrochemical performance degradation of the electrode caused by the mixture of additives or binders and active materials, resulting in the phenomenon of “dead capacitance” [37,38]. W Ouyang et al. prepared the three-dimensional porous structure of cellulose and restore oxidation graphene composite by ball milling assisted chemical reduction of graphene oxide, and it had potential applications in supercapacitors [39]. Graphene-cellulose nanocrystals aerogels prepared by Zhang et al. through two-step reduction and atmospheric pressure drying have excellent adsorption properties and mechanical strength [40].

In order to effectively solve the problem of graphene agglomeration, the electrical properties of graphene are maximized to prepare electrode materials for supercapacitors with excellent quality. In this work, a new preparation method of graphene-cellulose composite aerogel was proposed. It is the combination of the three-dimensional structure of graphene prepared by hydrothermal reduction method and the insertion of spacers between graphene sheets. Firstly, the graphene-cellulose composite hydrogel was prepared by hydrothermal method in the mixed solution of GO and cellulose, and then, the graphene-cellulose composite aerogel was prepared by freeze drying. The specific capacitance of the composite aerogel can reach 202 F/g at 5 mA/cm^2^ scanning rate. Lightweight composites can also withstand about 1095 times their weight without breaking. Effective spacer selection and simple and fast preparation methods make this porous, light, mechanically strong, high capacitance and environmentally friendly three-dimensional aerogel structure have great potential for supercapacitor electrode materials.

## 2. Results

### 2.1. Microstructure and Chemical Characterization

#### 2.1.1. SEM

**Figure 1:**
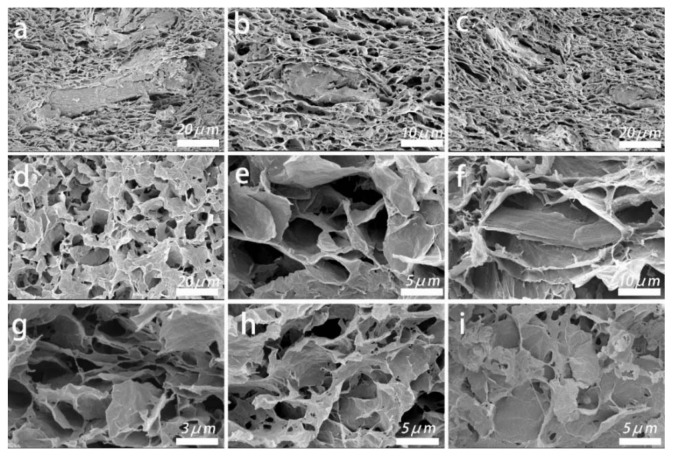
SEM images of (**a**) GA-MCC-3 aerogel, (**b**) GA-MCC-4 aerogel, (**c**) GA-MCC-5 aerogel, (**d**,**e**) GA aerogel in different magnifications, (**f**,**i**) GA-MCC-1 aerogel in different magnifications, (**g**,**h**) GA-MCC-2 aerogel in different magnifications.

As shown by SEM, all samples exhibit uniform three-dimensional porous structure. From Figure 1a–i, it can be seen that there is a close connection between cellulose and graphene. With the increase of cellulose content, the number of pores increases, and the pore wall becomes thicker. From Figure 1a–d, it can be seen that the number of pores in graphene-cellulose aerogels is much more than that of graphene aerogels under the same scanning times, which may be due to the support of cellulose in the composites. From Figure 1h,i, it can be seen that the tight connection between cellulose and graphene is wire-drawing, which helps to improve the mechanical properties of the composites. The smaller the cellulose content is, the clearer the edge of the pore wall is, which is caused by the stronger electron scattering of graphene than that of cellulose.

#### 2.1.2. TEM

**Figure 2:**
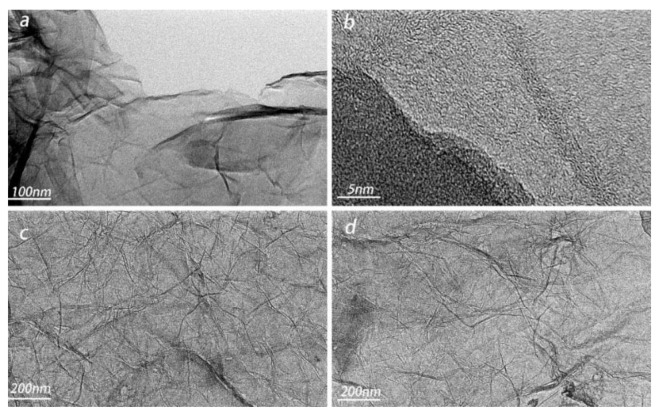
TEM images of (**a**,**b**) GA aerogel and (**c**,**d**) GA-MCC-4 aerogel.

From Figure 2a, it can be seen that the GA aerogel layer has a more transparent place, and the edge of the transparent part has darker stripes, which is the cross-linked place of the graphene aerogel layer, indicating the formation of a spatial three-dimensional cross-linked network structure. In Figure 2a, there are winding folds running through the graphene sheets, which is due to the folding structure of the graphene sheets of two-dimensional nanomaterials are the main source of micropores/mesopores. Figure 2b shows flat, regular graphene sheets overlap or overlap fold, lattice is a typical hexagonal polycrystalline electron diffraction pattern. From Figure 2c,d, which is the TEM diagram of GA-MCC-4 aerogel, cellulose is evenly distributed around graphene, forming a network structure, indicating that cellulose is closely connected with graphene, which also makes a microscopic explanation for the improvement of mechanical properties of the composite aerogel.

#### 2.1.3. FTIR

**Figure 3:**
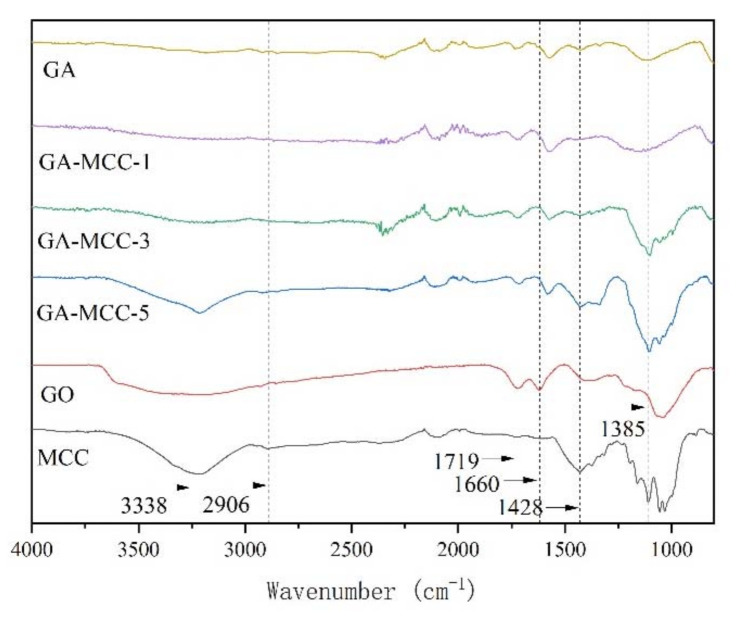
FTIR spectra of GA aerogel, GA-MCC-1 aerogel, GA-MCC-3 aerogel, GA-MCC-5 aerogel, GO and MCC.

Figure 3 is the infrared spectra of GA-MCC aerogel, GA aerogel, GO and MCC. For the curve of GO, it exhibits four characteristic bands at 3338 cm^−1^, 1719 cm^−1^, 1660 cm^−1^, and 1385 cm^−1^ correspond to O-H, C=O, C=C, and C-OH vibrations indicating the presence of hydroxyl, carbonyl, and benzene carboxyl groups bounded to the basal and edges of GO, respectively. In the FTIR spectra of GA aerogels and GA-MCC aerogels, these bands weakened. Even at 3338 cm^−1^ and 1660 cm^−1^ positions, there is almost no band. This indicates that GO has been reduced to GA [41,42,43,44]. In the FTIR spectra of MCC curve and GA-MCC aerogel, 3338 cm^−1^ and 1428 cm^−1^ vibration bands represent O-H and C-H bonds, respectively, which are characteristic bands of cellulose [45]. From the GA-MCC-1, GA-MCC-3, GA-MCC-5, and MCC curves, it can be observed that the bands of O-H bond at 3338 cm^−1^ and C-H bond at 1428 cm^−1^ increase in turn. It can be concluded that with the increase of cellulose content in raw materials, the number of O-H and C-H bonds in the composites increases gradually. O-H bond and C-H bond can form hydrogen bond, so that the mechanical strength of the composites increases gradually, which also provides the basis for the mechanical properties test results later.

#### 2.1.4. XRD

**Figure 4:**
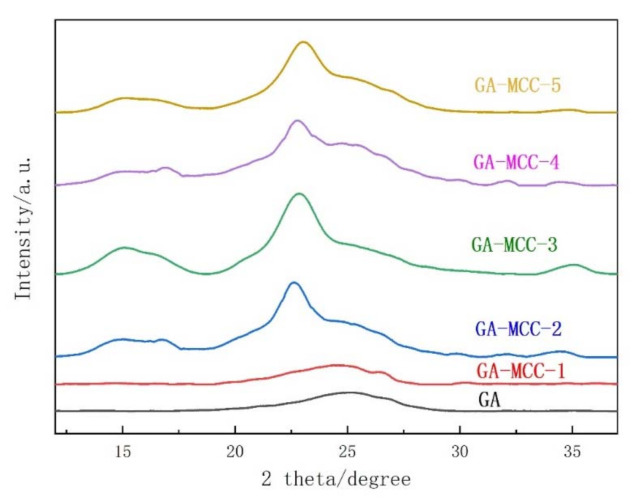
XRD of GA aerogel and GA-MCC (1–5) aerogels.

Figure 4 is the XRD pattern of graphene aerogel and graphene-cellulose composite aerogel. It can be seen from the Figure 4 that with the increase of cellulose content, the peaks of GA-MCC-2 to GA-MCC-5 samples fluctuate around 14–16°, which is the crystal structure feature of cellulose I [46]. GA and GA-MCC-1 showed a diffraction peak at 25.5°, which was the characteristic peak of graphene, indicating that graphene content was high. The (001) and (002) crystal planes of graphene oxide often correspond to 11.7° and 11.2° peaks that do not appear in all samples, indicating that graphene oxide has been completely transformed into graphene [47,48]. Although GO removed a large number of oxygen-containing functional groups in the reduction process, the graphene nanosheets were restacked under the action of π–π bond, but with the increase of cellulose content, the peak near 25.5° of GA-MCC-1 shifted to the left. According to the Bragg equation, the spacing of graphene sheets was increasing, which indicated that cellulose had been successfully inserted between graphene sheets, further indicating that the addition of cellulose can reduce the π–π stacking between graphene sheets, which also makes the further enhancement of mechanical and electrical properties of microcomposite aerogels explain.

#### 2.1.5. BET

**Figure 5:**
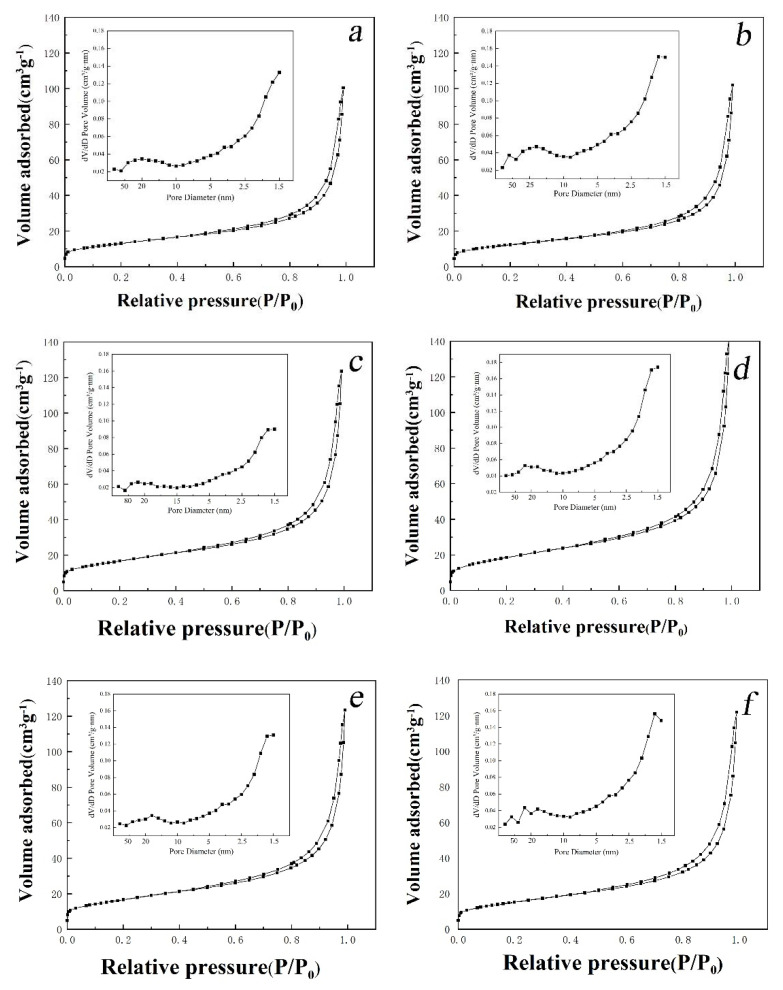
Pore characterization of GA aerogel and GA-MCC aerogels. The nitrogen adsorption-desorption curves and BJH pore distribution curves of (**a**) GA aerogel and (**b**–**f**) GA-MCC-1 to GA-MCC-5 aerogels.

The specific surface area and pore structure of GA aerogel and graphene-cellulose composite aerogel were determined by nitrogen adsorption-desorption test. As shown in Figure 5, the obvious type IV physical adsorption isotherm shows that all the samples show typical mesoporous structure. The formation of H3 hysteresis loop is due to the gap between the mesopores and the wrinkled wide gap accumulated in the graphene layer. These gaps lead to the eruption of nitrogen in the desorption process [49].

As shown in Table 1, with the increase of cellulose content, the specific surface area of composite aerogels increased first and then decreased. The increase of the specific surface area of the composite aerogel is attributed to the addition of MCC. The connection between MCC and GA makes the pore structure of the composite aerogel more compact. However, when the content of MCC is too high, it will cause the collapse of the pore structure of the composite aerogel, which makes the specific surface area of the composite aerogel decrease slightly. The GA-MCC-4 aerogel has the largest specific surface area. Therefore, GA-MCC-4 aerogels were selected for subsequent electrical properties measurement. This porous surface morphology is beneficial for the application of supercapacitors, because the porous structure can provide more active sites for ion accumulation.

### 2.2. Mechanical Property

**Figure 6:**
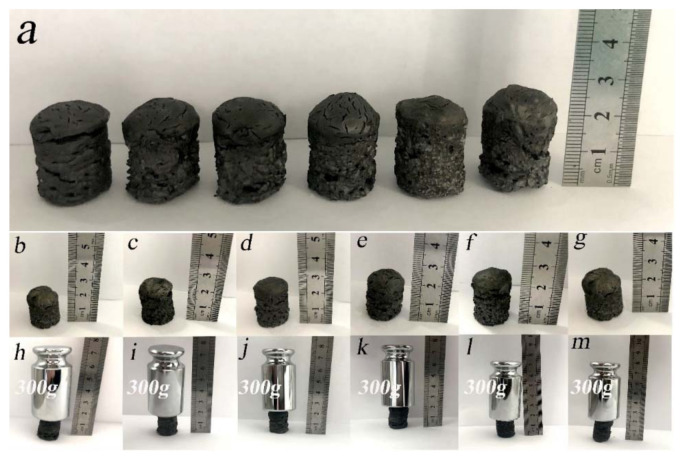
Compression test pictures of GA aerogel and GA-MCC (1–5) aerogel. (**a**) Six groups of aerogel samples. (**b**–**g**) GA aerogel and GA-MCC (1–5) aerogel. (**h**–**m**) GA aerogel and GA-MCC (1–5) aerogel subjected to a mass pressure of 300 g.

**Figure 7:**
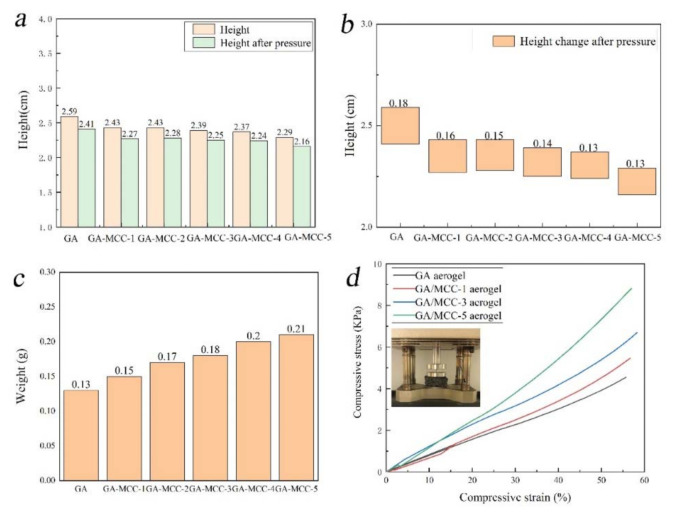
(**a**) Height diagram, (**b**) height change diagram after pressure, (**c**) weight diagram, and (**d**) stress–strain curve of GA aerogel and GA-MCC (1–5) aerogel.

Figure 6 shows the top compression test of GA aerogel and GA-MCC aerogel. Figure 7a–c shows the detailed physical parameters of GA aerogel and GA-MCC aerogel after compression. As shown in Figure 6, all aerogels are not broken when subjected to 300 g mass. There is no significant change in the height after compression, which shows excellent compressive properties. Figure 7 shows that with the increase of cellulose content, the quality of GA-MCC aerogels is gradually improved, the density is gradually increased, the height is reduced, and the height change after compression is gradually reduced, which shows that some cellulose has been successfully dispersed between graphene sheets and formed a good combination between them. This is also confirmed in the previous SEM, TEM, XRD, and FTIR test results. The samples were subjected to DMA compression test at room temperature. The test results are shown in Figure 7d. It can be seen from the stress–strain curve that with the increase of cellulose content, the composite aerogels showed better mechanical properties. GA-MCC-5 aerogels can withstand up to 9.11 KPa, equivalent to 1095 times the weight of self-weight, much higher than the maximum pressure capacity of GA aerogels (4.34 KPa). GA-MCC aerogels have higher pressure capacity than similar density aerogels prepared by Mi and Zhao [50,51]. The maximum pressure was also higher than that of graphene/cellulose nanocrystals aerogel (6 KPa) prepared by Zhang et al. through two-step reduction process and atmospheric drying method [40]. It can be seen that due to the addition of cellulose, the pressure-bearing capacity of the composite increased, indicating that cellulose played a supporting role and enhanced graphene.

### 2.3. Electrochemical Performance

**Figure 8:**
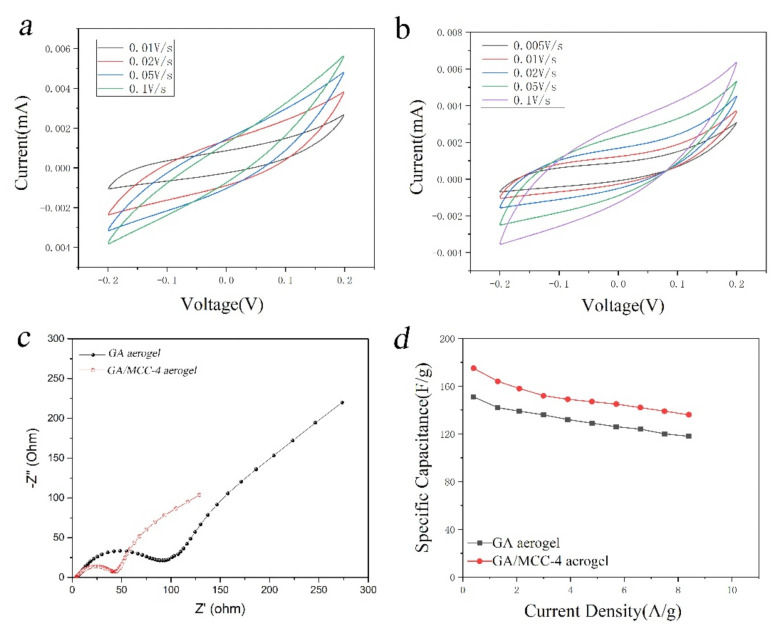
Comparison of GA aerogel and GA-MCC-4 aerogel in a three-electrode system with 6 M KOH as the electrolyte. (**a**) CV curves of GA aerogel at different scan rates. (**b**) CV curves of GA-MCC-4 aerogel at different scan rates. (**c**) The Nyquist impedance plots of the GA aerogel and GA-MCC-4 aerogel. (**d**) Specific capacitance retention of GA aerogel and GA-MCC-4 aerogel.

In order to evaluate the electrochemical behavior of the samples, electrochemical tests were carried out on GA aerogel and GA-MCC-4 aerogel. The electrical properties were evaluated by cyclic voltammetry and AC impedance method. Figure 8a,b shows CV curves of GA aerogel GA -MCC-4 aerogel with potential window of −0.2–0.2 V at different scanning rates. These CV curves show ideal capacitance behavior, and the image shape is close to rectangle. With the increase of scanning rate, the area of CV curve of all samples increased. Both of them can be maintained at a high scanning rate of up to 100 mA/s, indicating that GA aerogel and GA-MCC-4 aerogel have excellent electrochemical properties. From the comparison in Figure 8a,b, it can be seen that the capacitance of GA-MCC-4 aerogel was larger than that of GA aerogel. This is due to the addition of cellulose in GA-MCC composite aerogels, although the resistance value will increase to a certain extent, but the graphene itself is prone to agglomeration caused by van der Waals force and π–π force, which makes the resistance value decrease, and the addition of cellulose will slow down the agglomeration between graphene. At the same time, since graphene nanosheets cannot avoid restacking, the diffusion of electrolyte ions in the dense stacking structure of GA nanosheets becomes difficult, and the specific surface area of graphene-based materials is significantly lower than the theoretical value (2600 m^2^/g). For GA-MCC composite aerogels, the cellulose in mesoporous can not only effectively improve the accessible surface area of GA and electrolyte but also significantly reduce the ion diffusion distance from electrolyte nanoreservoir to GA nanosheets. Therefore, the capacitance performance of GA-MCC-4 aerogel is slightly increased compared with GA aerogel. According to the CV curve and specific capacitance calculation formula, the specific capacitance of GA-MCC-4 aerogel can reach 202 F/g at the scanning rate of 5 mA/cm^2^. At the scanning rate of 10 mA/cm^2^, the specific capacitances of GA aerogel and GA-MCC-4 aerogel were 91 F/g and 147 F/g, respectively. According to the previous literature, Lamberti, A et al. also used hydrothermal reduction method to prepare graphene and copper composite aerogel. The highest specific capacitance was 62.3 F/g at 5 mA/cm^2^ scanning rate [52]. The reduced graphene oxide-cellulose hybrid aerogels prepared by ball milling reduction method by Ouyang W et al. had the highest specific capacitance of 71.2 F/g at the scanning rate of 5 mA/cm^2^ [36]. The reduced graphene oxide-cellulose hybrid aerogels prepared by ball milling reduction method by Ouyang W et al. had the highest specific capacitance of 71.2 F/g at the scanning rate of 5 mA/cm^2^ [39].

Figure 8c is the Nyquist plot of GA aerogel and GA-MCC-4 aerogel. It can be seen that the curve overlap is very good, indicating that the sample has good electrochemical cycle stability. At high frequency, the impedance curve is a typical semicircle. Since the intercept of the semicircle on the solid axis is expressed as its internal resistance, it is shown in the figure that the internal resistance of GA-MCC-4 aerogel decreases due to the presence of cellulose, because hydrophilic cellulose can enhance the contact between the electrolyte and GA-MCC-4 aerogel. This point can also be seen from Figure 8d, because although GA aerogels and GA-MCC-4 aerogels have similar specific surface area and mesopore structure but with the increase of current density, GA-MCC-4 aerogels show better capacitance performance, which shows that the presence of cellulose plays a role in slowing down the rate of capacitance decay. GA-MCC-4 aerogel combines porous structure with high conductivity to prepare supercapacitors with excellent capacitance, conduction rate, and stability, which has potential applications as electrode materials for supercapacitors.

## 3. Materials and Methods

### 3.1. Materials and Chemicals

Graphite crystal (99 wt.%) was purchased from Jiangsu Changde High-tech Carbon Materials Co., Ltd. (Changde, China). Sodium nitrate (NaNO_3_) was purchased from Shanghai Yixin Chemical Co., Ltd. (Shanghai, China). Potassium permanganate powder (KMnO_4_) was purchased from Qufu Xinxin Chemical Co., Ltd. (Qufu, China). Sulfuric acid (H_2_SO_4_ 98 wt.%) and hydrogen peroxide (H_2_O_2_ 30 wt.%) were purchased from Anshan Anji Chemical Co., Ltd. (Anshan, China). Deionized water was purchased from Sigma-Aldrich (Harbin, China). Microcrystalline cellulose (MCC) powder (particle size: 50 μm) was purchased from Shanghai Aladdin Biochemical Technology Co., Ltd. (Shanghai, China).

### 3.2. Preparation of GO

Firstly, 46 mL concentrated sulfuric acid was cooled to 4 °C in ice bath. Under the action of magnetic stirrer, 2 g graphite powder, 1 g sodium nitrate, and 6 g potassium permanganate were slowly added to concentrated sulfuric acid. Control solution temperature at 5–10 °C and keep 90 min. Then, the mixed solution was transferred to a water bath to maintain it at 35–40 °C for intermediate temperature reaction. Take out the mixed solution 30 min later. Then, 92 mL deionized water was added drop by drop into the above reaction solution. In the reaction process, the water addition rate and amount should be controlled to keep the temperature of the reaction solution at about 95 °C. When the solution changes from dark green to red-brown, the high temperature reaction ends. A 30 wt.% hydrogen peroxide solution was added to the solution until no obvious bubbles were generated. At this time, the solution immediately turned into golden yellow and then turned into brownish yellow. The obtained solution was ultrasoniced for 30 min, and precipitated, centrifuged and put through dialysis to obtain high purity graphene oxide solution.

### 3.3. Preparation of GA-MCC Aerogel

GO (200 mg) was dissolved in 20 mL deionized water to prepare 10 mg/mL GO solution. Six groups of 10 mg/mL GO solution were added 0, 20, 50, 100, 150, and 200 mg cellulose, stirring at room temperature for 2 h, until the solution mixed evenly. Pour six groups of solution into the hydrothermal reactor, and then put the hydrothermal reactor into the oven. Graphene hydrogel and five groups of graphene-cellulose hydrogel were obtained by heating at 180 °C for 10 h. All samples were frozen rapidly in liquid nitrogen and then put into the freeze dryer. After 48 h, graphene aerogel and graphene-cellulose aerogel were obtained. They were named GA, GA-MCC-1, GA-MCC-2, GA-MCC3, GA-MCC-4, and GA-MCC-5, respectively, and further characterization experiments were carried out.

### 3.4. Characterization

The scanning electron microscope (SEM) images were obtained by using QUANTA200 instrument. Transmission electron microscopy (TEM) was measured by transmission electron microscopy (JEM-2100). Fourier transform infrared spectroscopy (FT-IR) was performed on Spectrum 400 instrument, and the scanning range was 550–4000 cm^−1^. X-ray diffraction (XRD) analysis was performed using XRD-6100 in the scanning range of 5–60° and the scanning speed of 5°/min. Brunner–Emmet–Teller (BET) measurement is to obtain the specific surface area and pore structure of aerogels by nitrogen adsorption. The dynamic thermal analyzer (DMA) with the model of DMA242E Artemis was used. The sample was cut into a cylinder with a height of 0.5 cm, and the compression test was carried out at room temperature. Electrochemical measurement was performed on an electrochemical workstation (1470 E), using 6 M potassium hydroxide solution as electrolyte, silver chloride electrode as reference electrode, graphite rod as counter electrode, platinum sheet electrode clip as working electrode. The aerogels were cut into small wafers with a height of 0.25 cm and a diameter of 1.8 cm, and one quarter was taken as the experimental material. The electrical properties were evaluated by cyclic voltammetry and AC impedance. The scan rate of CV curve was 5, 10, 20, 50, and 100 mA/s, and the potential window was −0.2–0.2 V. The electrochemical impedance spectroscopy (EIS) curves were collected from 10 mHz to 100 kHz.

## 4. Conclusions

In this work, graphene-cellulose hydrogels were prepared by one-step hydrothermal method, and then freeze-dried to prepare graphene-cellulose aerogels. The surface morphology, internal composition, mechanical and electrical properties of the composite aerogels were obtained by SEM, TEM, XRD, FTIR, BET, mechanical property test, and electrical property test. SEM, TEM, and BET tests showed that the composite had uniform mesoporous structure and large specific surface area, and the cellulose and graphene had been mixed evenly. The results of XRD and FTIR confirmed that there was a close hydrogen bond between cellulose and graphene, and cellulose had been successfully inserted between graphene sheets to slow down its π–π stacking. Through the pressure test of composite aerogels, it was found that the light composite aerogels could withstand the weight of about 1095 times of their own weight without breaking. Moreover, with the increase of cellulose content, the pressure value of composite aerogels increased. It shows that cellulose plays a supporting role in the composite. When the electrical properties of the composite were tested, the specific capacitance of the composite aerogel could reach 202 F/g at the scanning rate of 5 mA/cm^2^. This lightweight, solid, high capacitance, green three-dimensional aerogel structure has great potential value as electrode material for supercapacitors.

## Figures and Tables

**Table 1:** Surface area of GA aerogel and GA-MCC-1 to GA-MCC-5 aerogels.

Sample	GA Aerogel	GA-MCC-1 Aerogel	GA-MCC-3 Aerogel	GA-MCC-4 Aerogel	GA-MCC-5 Aerogel
Surface area (m^2^/g)	76.31	84.24	96.57	94.18	84.24

## Data Availability

The data that support the findings of this study are available from the corresponding author upon reasonable request.

## Sample Availability

Samples of the compounds (GA aerogel, GA-MCC-1 aerogel, GA-MCC-2 aerogel, GA-MCC-3 aerogel, GA-MCC-4 aerogel, and GA-MCC-5 aerogel) are available from the authors.

## References

[1] Wang F., Wu X., Yuan X., Liu Z., Zhang Y., Fu L., Zhu Y., Zhou Q., Wu Y., Huang W. (2017). Latest advances in supercapacitors: From new electrode materials to novel device designs. Chem. Soc. Rev..

[2] Zhang L.L., Zhao X.S. (2009). Carbon-based materials as supercapacitor electrodes. Chem. Soc. Rev..

[3] Lin X.J., Wu Z.M., Zhang C.Y., Liu S.J., Nie S.X. (2018). Enzymatic pulping of lignocellulosic biomass. Ind. Crops Prod..

[4] Fan J., Li T., Ren Y.Z., Qian X.R., Wang Q.W., Shen J., Ni Y.H. (2017). Interaction between two oppositely charged starches in an aqueous medium containing suspended mineral particles as a basis for the generation of cellulose-compatible composites. Ind. Crop. Prod..

[5] Qian Y.Q., Ismail I.M., Stein A. (2014). Ultralight, high-surface-area, multifunctional graphene-based aerogels from self-assembly of graphene oxide and resol. Carbon.

[6] Kanninen P., Luong N.D., Sinh L.H., Anoshkin I.V., Tsapenko A., Seppala J., Nasibulin A.G., Kallio T. (2016). Transparent and flexible high-performance supercapacitors based on single-walled carbon nanotube films. Nanotechnology.

[7] Kim T.Y., Lee H.W., Stoller M., Dreyer D.R., Suh K.S. (2011). High-performance supercapacitors based on poly(ionic liquid)-modified graphene electrodes. Acs Nano.

[8] Chen J., Li C., Shi G.Q. (2013). Graphene Materials for Electrochemical Capacitors. J. Phys. Chem. Lett..

[9] Ambrosi A., Chua C.K., Bonanni A., Pumera M. (2014). Electrochemistry of graphene and related materials. Chem. Rev..

[10] Cao X.H., Yin Z.Y., Zhang H. (2014). Three-dimensional graphene materials: Preparation, structures and application in supercapacitors. Energy Environ..

[11] Zhu Y.F., Huang H.F., Zhou W.Z., Li G.X., Liang X.Q., Guo J., Tang S.L. (2017). Low temperature reduction of graphene oxide film by ammonia solution and its application for high-performance supercapacitors. J. Mater. Sci. Mater. Electron..

[12] Walsh E.D., Han X.G., Lacey S.D., Kim J.W., Connell J.W., Hu L.B., Lin Y. (2016). Dry-processed, binder-free holey graphene electrodes for supercapacitors with ultrahigh areal loadings. ACS Appl. Mater. Interfaces.

[13] Gao Y.D., Zhang Y.Y., Zhang Y., Xie L.J., Li X.M., Su F.Y., Wei X.X., Xu Z.W., Chen C.M., Cai R. (2016). Three-dimensional paper-like graphene framework with highly orientated laminar structure as binder free supercapacitor electrode. J. Energy Chem..

[14] Wu Z.S., Sun Y., Tan Y.Z., Yang S.B., Feng X.L., Mullen K. (2012). Three-Dimensional Graphene-Based Macro- and Mesoporous Frameworks for High-Performance Electrochemical Capacitive Energy Storage. J. Am. Chem. Soc..

[15] Chen W.S., Yu H.P., Liu Y.X. (2011). Preparation of millimeter-long cellulose I nanofibers from bamboo fibers. Carbohydr. Polym..

[16] Wang Z.J., Zhou X.Z., Zhang J., Boey F., Zhang H. (2009). Direct electrochemical reduction of single-layer graphene oxide and subsequent functionalization with glucose oxidase. J. Phys. Chem. C.

[17] Chen W., Xia C., Alshareef H.N. (2015). Graphene based integrated tandem super capacitors fabricated directly on separators. Nano Energy.

[18] Xu Y.X., Sheng K.X., Li C., Shi G.Q. (2010). Self-assembled graphene hydrogel via a one-step hydrothermal process. ACS Nano.

[19] Tang Z.H., Shen S.L., Zhuang J., Wang X. (2010). Noble-metal-promoted three-dimensional macroassembly of single-layered graphene oxide. Angew. Makromol. Chem..

[20] Jiang X., Ma Y.W., Li J.J., Fan Q.L., Huang W. (2010). Self-Assembly of Reduced Graphene Oxide into Three-Dimensional Architecture by Divalent Ion Linkage. J. Phys. Chem. C.

[21] Zhang L., Shi G.Q. (2011). Preparation of Highly Conductive Graphene Hydrogels for Fabricating Supercapacitors with High Rate Capability. J. Phys. Chem. C.

[22] Jiang Q.S., Kacica C., Soundappan T., Liu K.K., Tadepalli S., Biswas P., Singamaneni S. (2017). An in situ grown bacterial nanocellulose/graphene oxide composite for flexible supercapacitors. J. Mater. Chem..

[23] Wu Z.S., Yang S.B., Sun Y., Parvez K., Feng X.L., Mullen K. (2012). 3D Nitrogen-Doped Graphene Aerogel-Supported Fe_3_O_4_ Nanoparticles as Efficient Eletrocatalysts for the Oxygen Reduction Reaction. J. Am. Chem. Soc..

[24] Li M.Z., Miao Y.Y., Zhai X.Y., Yin Y.X., Zhang Y.T., Jian Z.B., Wang X.Y., Sun L.P., Liu Z.B. (2019). Preparation of and research on bioinspired graphene oxide-nanocellulose-polydopamine ternary artificial nacre. Mater. Des..

[25] Li M.Z., Wang X.Y., Zhao R., Miao Y.Y., Liu Z.B. (2021). A novel graphene-based micro-nano architecture with high strength and conductivity inspired by multiple creatures. Sci. Rep..

[26] Nie S.X., Zhang K., Lin X.J., Zhang C.Y., Yan D.P., Liang H.M., Wang S.F. (2018). Enzymatic pretreatment for the improvements of dispersion and film properties of cellulose nanofibrils. Carbohydr. Polym..

[27] Zhang K., Zhang Y.H., Yan D.P., Zhang C.Y., Nie S.X. (2018). Enzyme-assisted mechanical production of cellulose nanofibrils: Thermal stability. Cellulose.

[28] Zhang X., Liu X., Zheng W., Jin Z. (2012). Regenerated cellulose/graphene nanocomposite films prepared in DAMC/LiCl solution. Carbohydr. Polym..

[29] Isogai A., Saito T., Fukuzumi H. (2011). TEMPO-oxidized cellulose nanofibers. Nanoscale.

[30] Li Y.Y., Zhu H.L., Shen F., Wan J.Y., Lacey S., Fang Z.Q., Dai H.Q., Hu L.B. (2015). Nanocellulose as green dispersant for two-dimensional energy materials. Nano Energy.

[31] Yao S.Q., Nie S.X., Zhu H.X., Wang S.F., Song X.P., Qin C.R. (2017). Extraction of hemicellulose by hot water to reduce adsorbable organic halogen formation in chlorine dioxide bleaching of bagasse pulp. Ind. Crop. Prod..

[32] Yang C., Li D.G. (2015). Flexible and foldable supercapacitor electrodes from the porous 3D network of cellulose nanofibers, carbon nanotubes and polyaniline. Mater. Lett..

[33] Ge D.T., Yang L.L., Fan L., Zhang C.F., Xiao X., Gogotsi Y., Yang S. (2015). Foldable supercapacitors from triple networks of macroporous cellulose fibers, single-walled carbon nanotubes and polyaniline nanoribbons. Nano Energy.

[34] Yao S.Q., Nie S.X., Yuan Y., Wang S.F., Qin C.R. (2015). Efficient extraction of bagasse hemicelluloses and characterization of solid remainder. Bioresour. Technol..

[35] Kang Y.J., Chun S.J., Lee S.S., Kim B.Y., Kim J.H., Chung H., Lee S.Y., Kim W. (2012). All-solid-state flexible supercapacitors fabricated with bacterial nanocellulose papers, carbon nanotubes, and triblock-copolymer ion gels. ACS Nano.

[36] Xing J.H., Tao P., Wu Z.M., Xing C.Y., Liao X.P., Nie S.X. (2019). Nanocellulose-graphene composites: A promising nanomaterial for flexible supercapacitors. Carbohydr. Polym..

[37] Shao Y.L., El-Kady M.F., Wang L.J., Zhang Q.H., Li Y.G., Wang H.Z., Mousavi M.F., Kaner R.B. (2015). Graphene-based materials for flexible supercapacitors. Chem. Soc. Rev..

[38] Sun Z.P., Popa D., Hasan T., Torrisi F., Wang F.Q., Kelleher E.J.R., Travers J.C., Nicolosi V., Ferrari A.C. (2010). A stable, wideband tunable, near transform-limited, graphene-mode-locked, ultrafast laser. ACS Nano.

[39] Ouyang W.Z., Sun J.H., Memon J., Wang C., Geng J.X., Huang Y. (2013). Scalable preparation of three-dimensional porous structures of reduced graphene oxide-cellulose composites and their application in supercapacitors. Carbon.

[40] Zhang X.F., Liu P., Duan Y.X., Jiang M., Zhang J.M. (2017). Graphene/cellulose nanocrystals hybrid aerogel with tunable mechanical strength and hydrophilicity fabricated by ambient pressure drying technique. RSC Adv..

[41] Yang H.P., Yan R., Chen H.P., Lee D.H., Zheng C.G. (2007). Characteristics of hemicellulose, cellulose and lignin pyrolysis. Fuel.

[42] Feng Y.Y., Zhang X.Q., Shen Y.T., Yoshino K., Feng W. (2012). A mechanically strong, flexible and conductive film based on bacterial cellulose/graphene nanocomposite. Carbohydr. Polym..

[43] Park S., Lee K.S., Bozoklu G., Cai W., Nguyen S.T., Ruoff R.S. (2008). Graphene oxide papers modified by divalent ions-enhancing mechanical properties via chemical cross-linking. ACS Nano.

[44] Meador M.A.B., Fabrizio E.F., Ilhan F., Dass A., Zhang G.H., Vassilaras P., Johnston J.C., Leventis N. (2005). Cross-linking amine-modified silica aerogels with epoxies: Mechanically strong lightweight porous materials. Chem. Mater..

[45] Kim H., Abdala A.A., Macosko C.W. (2015). Graphene/polymer nanocomposites. Macromolecules.

[46] Gao K.Z., Shao Z.Q., Li J., Wang X., Peng X.Q., Wang W.J., Wang F.J. (2013). Cellulose nanofiber-graphene all solid-state flexible supercapacitors. J Mater. Chem. A.

[47] Stobinski L., Lesiak B., Malolepszy A., Mazurkiewicz M., Mierzwa B., Zemek J., Jiricek P., Bieloshapka I. (2014). Graphene oxide and reduced graphene oxide studied by the XRD, TEM and electron spectroscopy methods. J. Electron Spectros. Relat. Phenom..

[48] Dang L.N., Seppala J. (2015). Electrically conductive nanocellulose/graphene composites exhibiting improved mechanical properties in high-moisture condition. Cellulose.

[49] Zhang M.Y., Sun Y.Y., Shi J.J., Ning W.S., Hou Z.Y. (2017). Selective glycerol oxidation using platinum nanoparticles supported on multi-walled carbon nanotubes and nitrogen-doped graphene hybrid. Chin. J. Catal..

[50] Mi H.Y., Jing X., Politowicz A.L., Chen E., Huang H.X., Turng L.S. (2018). Highly compressible ultra-light anisotropic cellulose/graphene aerogel fabricated by bidirectional freeze drying for selective oil absorption. Carbon.

[51] Zhao S.M., Yan Y.H., Gao A.L., Zhao S., Cui J., Zhang G.F. (2018). Flexible Polydimethylsilane Nanocomposites Enhanced with a Three-Dimensional Graphene/Carbon Nanotube Bicontinuous Framework for High-Performance Electromagnetic Interference Shielding. ACS Appl. Mater. Interfaces.

[52] Lamberti A., Gigot A., Bianco S., Fontana M., Castellino M., Tresso E., Pirri C.F. (2016). Self-assembly of graphene aerogel on copper wire for wearable fiber-shaped supercapacitors. Carbon.

